# InContext: curation of medical context for drug indications

**DOI:** 10.1186/s13326-021-00234-4

**Published:** 2021-02-12

**Authors:** Kody Moodley, Linda Rieswijk, Tudor I. Oprea, Michel Dumontier

**Affiliations:** 1 Institute of Data Science, Maastricht University, Paul-Henri Spaaklaan 1, 6229 GT Maastricht, The Netherlands; 2 Translational Informatics Division, Department of Internal Medicine, MSC09-5025, One University of New Mexico, Albuquerque, New Mexico 87131 USA; 3 UNM Comprehensive Cancer Center, 1201 Camino de Salud, Albuquerque, New Mexico 87102 USA

**Keywords:** Ontologies, Drug repurposing, Drug indications, Semantic similarity, Data quality

## Abstract

Accurate and precise information about the therapeutic uses (indications) of a drug is essential for applications in drug repurposing and precision medicine. Leading online drug resources such as DrugCentral and DrugBank provide rich information about various properties of drugs, including their indications. However, because indications in such databases are often partly automatically mined, some may prove to be inaccurate or imprecise. Particularly challenging for text mining methods is the task of distinguishing between general disease mentions in drug product labels and actual indications for the drug. For this, the qualifying medical context of the disease mentions in the text should be studied. Some examples include contraindications, co-prescribed drugs and target patient qualifications. No existing indication curation efforts attempt to capture such information in a precise way. Here we fill this gap by presenting a novel curation protocol for extracting indications and machine processable annotations of contextual information about the therapeutic use of a drug. We implemented the protocol on a reference set of FDA-approved drug product labels on the DailyMed website to curate indications for 150 anti-cancer and cardiovascular drugs. The resulting corpus - InContext - focuses on anti-cancer and cardiovascular drugs because of the heightened societal interest in cancer and heart disease. In order to understand how InContext relates with existing reputable drug indication databases, we analysed it’s overlap with a state-of-the-art indications database - LabeledIn - as well as a reputable online drug compendium - DrugCentral. We found that 40% of indications sampled from DrugCentral (and 23% from LabeledIn) respectively, could not be accounted for in InContext. This raises questions about the veracity of indications not appearing in InContext. The additional contextual information curated by InContext about disease mentions in drug SPLs provides a foundation for more precise, structured and formal representations of knowledge related to drug therapeutic use, in order to increase accuracy and agreement of drug indication extraction methods for in silico drug repurposing.

## Introduction

The growing costs and development times associated with drug manufacturing (estimated at 2.6 billion dollars and 10 years respectively per drug [1, 2]) have propelled advances in *drug repurposing*: discovering therapeutic uses (which, after regulatory approval are referred to as *indications*) for drugs that are different to the ones stated on their approved drug labels.

A critical requirement for many drug repurposing methods that predict novel indications for drugs, based on disease similarity and known drug – disease associations, is accurate and precise information about these drugs, diseases and associations [3].

There are various initiatives such as MEDI [4], Drugbank [5] and DrugCentral [6, 7] which aim to provide high quality, structured information about the therapeutic usage of drugs. However, because the indications in these resources are partly mined from literature in an automated fashion, their quality is often inconsistent [8–10]. Indeed, the proper capture of therapeutic intent in a machine-actionable format, suitable for automated medical reasoning and drug repurposing, is likely to require the development of a new formal model as an application ontology [11].

**Fig. 1:**
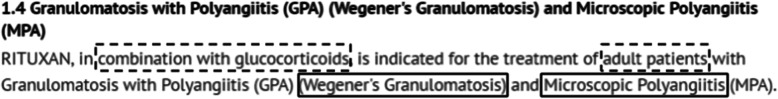
Example of important context information for an indication in an SPL (Drug: Rituximab)

These shortcomings are, in part, due to the fact that natural language processing (NLP) techniques used to extract these indications from unstructured text still face difficulties in terms of (i) recognizing disease mentions in text, (ii) separating irrelevant disease mentions in text from the relevant indications for a drug, and (iii) normalization of diseases to standard terms in application ontologies [12, 13]. Tasks (i) and (ii) are especially challenging because there are currently no machine processable ways to precisely represent contextual information from text about the therapeutic use of drugs. Figure 1 depicts an annotated snippet from a structured product label (SPL) from the *DailyMed* website for the anti-cancer drug *Rituximab* [14]. The text is taken from the section entitled “1 INDICATIONS AND USAGE” on the SPL webpage. DailyMed is a website which provides U.S. Food and Drug Administration (FDA)-approved digital SPLs for tens of thousands of drug products. Each SPL contains information such as indications, contraindications, adverse reactions, dosage etc. presented in unstructured free text.

From the text in Fig. 1 it is clear that *Rituxan* (the product which contains the active ingredient *Rituximab*) is indicated to treat *Wegener’s Granulomatosis (GPA)* and *Microscopic Polyangiitis (MPA)*. However, there is some medical context to these indications. The drug is only indicated for these diseases when used *in combination* with another class of drugs called *glucocorticoids.* Even then, it is only advocated for use in *adult patients*. Whereas a naïve text mining algorithm might directly associate Rituximab with GPA and MPA, it is important to be more precise in capturing the medical qualifiers for the drug’s therapeutic use. This is so that higher precision drug - disease associations are fed into drug repurposing algorithms which, in turn, may yield higher quality predictions.

Since online drug information databases are also known to partly automatically mine indications from drug product labels, it can lead to imprecise information in these sources which are known to be used in drug repurposing algorithms [15]. For example, in DrugCentral, *Poisoning by digitalis glycoside* is listed as an indication for the drug *Digoxin.* However, this is inaccurate because an overdose of Digoxin is a known *cause* of Poisoning by digitalis glycoside [16]. Another type of problem is imprecision of indications. For example, the use of *Alprostadil* is approved by the FDA to “maintain the patency of the *ductus arteriosus* until corrective or palliative surgery can be performed in *neonates* who have *congenital heart defects*” [17]. However, DrugCentral actually lists some of these congenital heart defects (e.g. *Pulmonary stenosis*, and *Tetralogy of Fallot*) as indications for this drug, which does not give a full description of when the drug should be administered.

Here we attempt to address the issue of imprecise capturing of medical context, by establishing a curation protocol for extracting drug indications together with their contextual therapeutic use information from FDA-approved SPLs. We use the protocol to curate a reference set of indications and medical context for a sample of drugs from the antineoplastic and cardiovascular classes (because of the heightened societal interest in heart disease and cancer). We call the resulting corpus *InContext.* InContext represents a novel attempt to curate therapeutic context for drug indications in a precise way for in silico drug repurposing research.

In order to get an initial impression of how InContext’s indications overlap with existing databases, we also performed a preliminary analysis of it’s overlap with 1) a leading online drug compendium - DrugCentral and 2) a state-of-the-art drug indications database - LabeledIn. We chose DrugCentral because it is regarded as one of the leading online drug information resources and attempts to distill most of the FDA-approved drug product information contained in *DailyMed* [18] in a computer-ready format. Similarly, LabeledIn is a state-of-the-art indications database which also uses DailyMed SPLs as its information source, which makes our comparison relevant.

The rest of this article is structured as follows: Section 2 gives some related work in drug indications extraction. Section 3 is the methods section which describes the InContext curation protocol, the corpus, the setup of our indications overlap study between InContext and two prominent drug indication databases - DrugCentral and LabeledIn. Section 4 discusses the results for calculating InContext’s overlap with DrugCentral and LabeledIn. Section 5 concludes the article by providing a summary of our key findings and plans for extending the investigation.

## Related work

To the best of our knowledge we are not aware of any other curations of therapeutic context information for medical drugs. However, there are numerous drug indication curation efforts [8–10, 12, 19, 20] and databases that have emerged over the last two decades. These include databases such as NDF-RT [21], MEDI [4] and SIDER [22]. There have also been attempts to assess overlap of indications in seven of these databases (including LabeledIn) [23]. The findings in this study were that there were many discrepancies among the indication sets stemming from issues with differing granularity in how indications were represented. DrugCentral has not been compared in overlap studies to date.

In terms of approaches to mine indications from text, the main steps are NER (named entity recognition) mapping of disease mentions in text and distinguishing between indications and irrelevant disease mentions. NER methods are largely accurate and there are established BioNLP tools to perform this step [20]. Recognition of drug indications is more challenging. There are two types of approaches to deal with this: human annotators (either biomedical experts [24] or crowdsourcing workers [19]) and machine learning classifiers [25]. The prediction performance of such automated drug indication extraction methods vary (For example MetaMap approach [9]: a recall of 95%, a precision of 77%, and an F score of 85% (based on training set of 6797 drug labels); Disease NER Tool trained on LabeledIn corpus approach [25]: a recall of 86%, a precision of 87% and an F score of 86% (based a training set of 500 drug labels). The most similar curation strategy to our own is the one employed by *LabeledIn* [24]. The purveyors of LabeledIn also mine SPLs on DailyMed for their indications and they also employ human annotators to disambiguate indications from general disease mentions in the text. However, there are two important distinctions between InContext and LabeledIn: 1) LabeledIn does not curate contextual information about the therapeutic use of a drug e.g. co-morbidities, contraindications and temporal usage aspects. This component is essential for our future endeavors to evaluate the quality of therapeutic usage information in online drug databases. 2) LabeledIn, in some cases, curates indications from multiple formulations (products) of the same drug, while InContext sources indications from one SPL (Fig. 3, Step 2).

LabeledIn is a more mature curation effort in terms of the number of drugs it documents. There are currently indications for over 8000 different drugs in the dataset, while InContext has just been established with annotations for 200 drugs. However, importantly, LabeledIn does not curate contextual information about the usage of drugs, which makes it difficult to determine the veracity of the drug-disease pairs without studying the accompanying SPL text manually. Obviously, disease terms alone are often not enough to capture the full conditions under which the drug may be administered.

## Methods (InContext - curation protocol and corpus)

In this section we supply details about the procedure for curating our FDA-approved reference set of drug indications and medical context.

### Selection of information source and drugs sample

We extracted our reference standard of accurate indications from SPLs on the DailyMed website. We choose the DailyMed resource because it distills US FDA-approved information (such as dosage, indications, contraindications, warnings and adverse reactions) about most marketed drugs in digitized, computer-readable product labels. DailyMed is the official provider of FDA label information and therefore provides trustworthy information. Finally, the machine-readable format of DailyMed SPLs makes it scalable to extract and analyze information content.

We decided to focus on antineoplastic and cardiovascular classes of drug because of the heightened societal interest in heart disease and cancer. We used the Anatomical Therapeutic Chemical Classification System (ATC) code prefixes of cardiovascular drugs (ATC codes of these drugs begin with the letter “C”) and antineoplastic drugs (ATC codes of these drugs begin with “L01”) to search for them in the DrugCentral SQL database. The search returned 800 drugs. We then wrote a computer script to programmatically search the DailyMed website for SPLs for these drugs (the steps can also be reproduced manually by using the DailyMed website search box). Execution of the script revealed that 200 of these drugs did not have listed SPLs on DailyMed (owing to the fact that they may be outdated, discontinued, withdrawn or approved by an organization other than the US FDA). This leaves us with 600 drug SPLs on DailyMed that have the potential to be annotated. We observed that manual annotation of a single SPL, by our human annotators, takes roughly between 5 and 20 min (the time varies according to the length of the text as well the density and specificity of medical terminology therein). This means that annotation of 600 drugs could in principle take 200 h to complete. With the limited financial and human resources we had at our disposal, we decided to start our annotation on a sample from the total list of 600. We randomly picked a sample of 200 drugs (33% of the total) conservatively estimated to take 67 h to annotate. This translated into a person-hour effort which was within our budgetary constraints. The sample used in this study is the subset of 158 drugs out of the 200 that are currently completed.

### Human annotators & software used in the protocol

To isolate relevant indications from the free text SPLs we had at our disposal, the expertise of 15 biomedical researchers and graduate students, from the Stanford Center for Biomedical Informatics Research and the Institute of Data Science at Maastricht University. The makeup of the team is described in more detail in Table 1.

**Table 1:** Expertise and qualifications of the members of the indication annotation team

Category	# of members	Expertise
Research professors	2	Biochemistry, Data Science & Translational medicine
Postdoctoral researchers	5	Drug repurposing, Molecular nutrition & Toxicogenomics, Bioinformatics & data quality, Clinical data science
PhD candidates	3	Biomedical Knowledge Graphs
Master students	5	Medical informatics, Statistics and Computer science

**Fig. 2:**
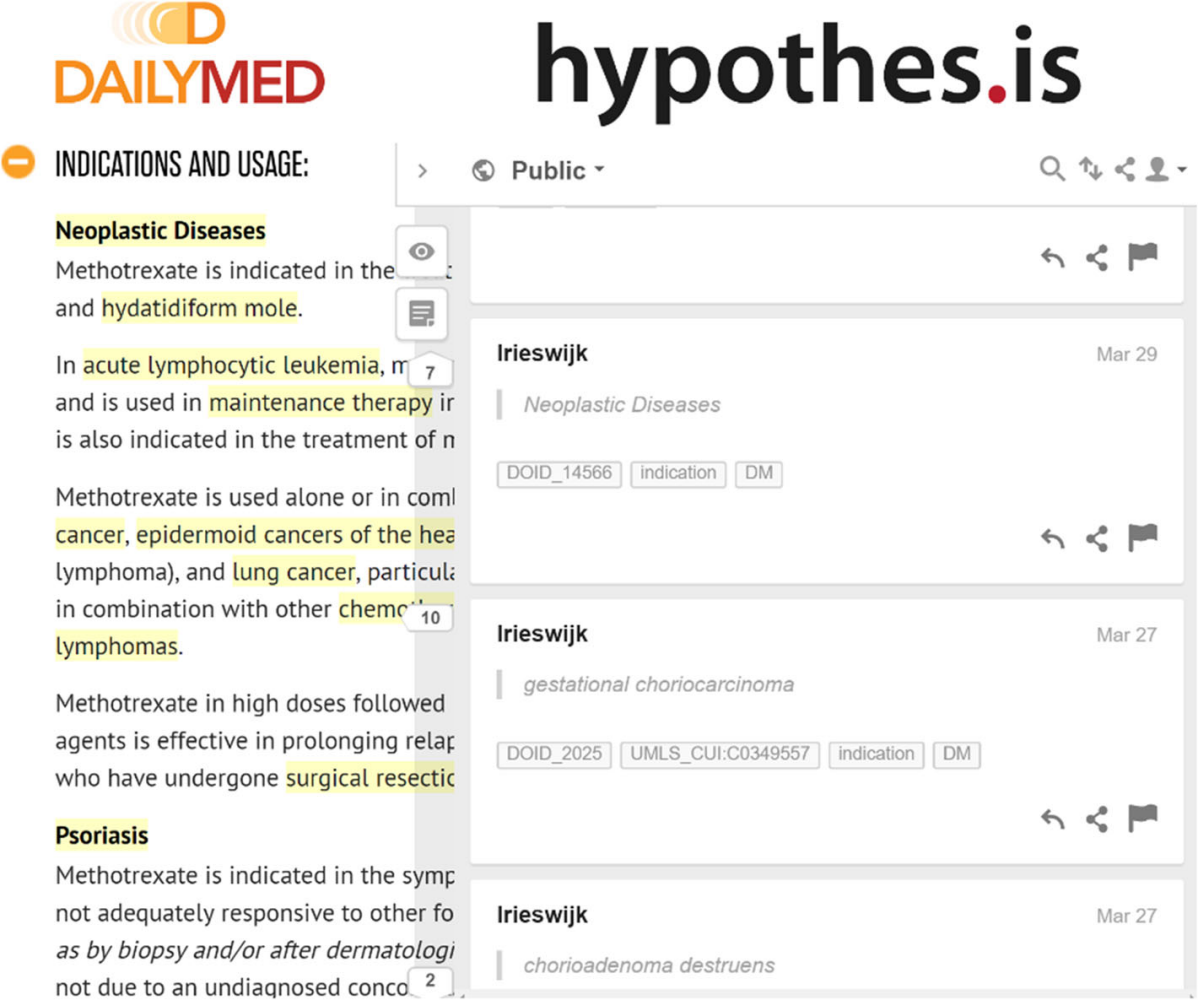
Hypothes.is browser plug-in example tagging of indications for an antineoplastic drug

Since indications are contained in free text descriptions within DailyMed web pages, formatted as SPLs, we decided to use a free and open-source web page annotator called *Hypothes.is* [26] to annotate the SPLs directly (see Fig. 2) with tags specifying indications and therapeutic use information. Hypothes.is is installed as a web browser plugin which allows the user to highlight text on any web page and annotate it with textual tags that can be accessed by other Hypothes.is users with the browser extension installed. The tags can also be extracted programmatically via the Hypothes.is public API [27]. To speed up the identification of factual indications in a particular SPL, we solicited the use of the National Center for Biomedical Ontologies (NCBO) *BioPortal annotator* [28]*.* BioPortal [29] itself is a well-known repository of biomedical ontologies maintained by the NCBO, while the annotator is a supplementary web service allowing one to automatically identify known bio-ontology terms in given free text. The BioPortal annotator is used in our annotation process to automate the recognition of diseases mentioned in the SPL. After this step, the information context of these disease mentions are analyzed by the human annotators to determine which of the mentions actually represent an indication for the drug. It should be noted that the BioPortal Annotator, and all other tools for identifying biomedical terms in text, do not have 100% precision or recall. MetaMap [30] is an alternative tool having lower precision but higher recall than the Annotator [31]. However, MetaMap is also shown to be orders of magnitude slower than the Annotator, and the latter is also capable of recognizing terms that MetaMap cannot [31]. Therefore, we motivate that the Annotator is a justifiable choice for this study. The annotation process occurred over a 9 month period from 2016 to 07-15 to 2017-04-13. The version change files for the ontology during this period can be downloaded in XML format from the BioPortal page for the Human Disease ontology [32].

### Annotation protocol

**Fig. 3:**
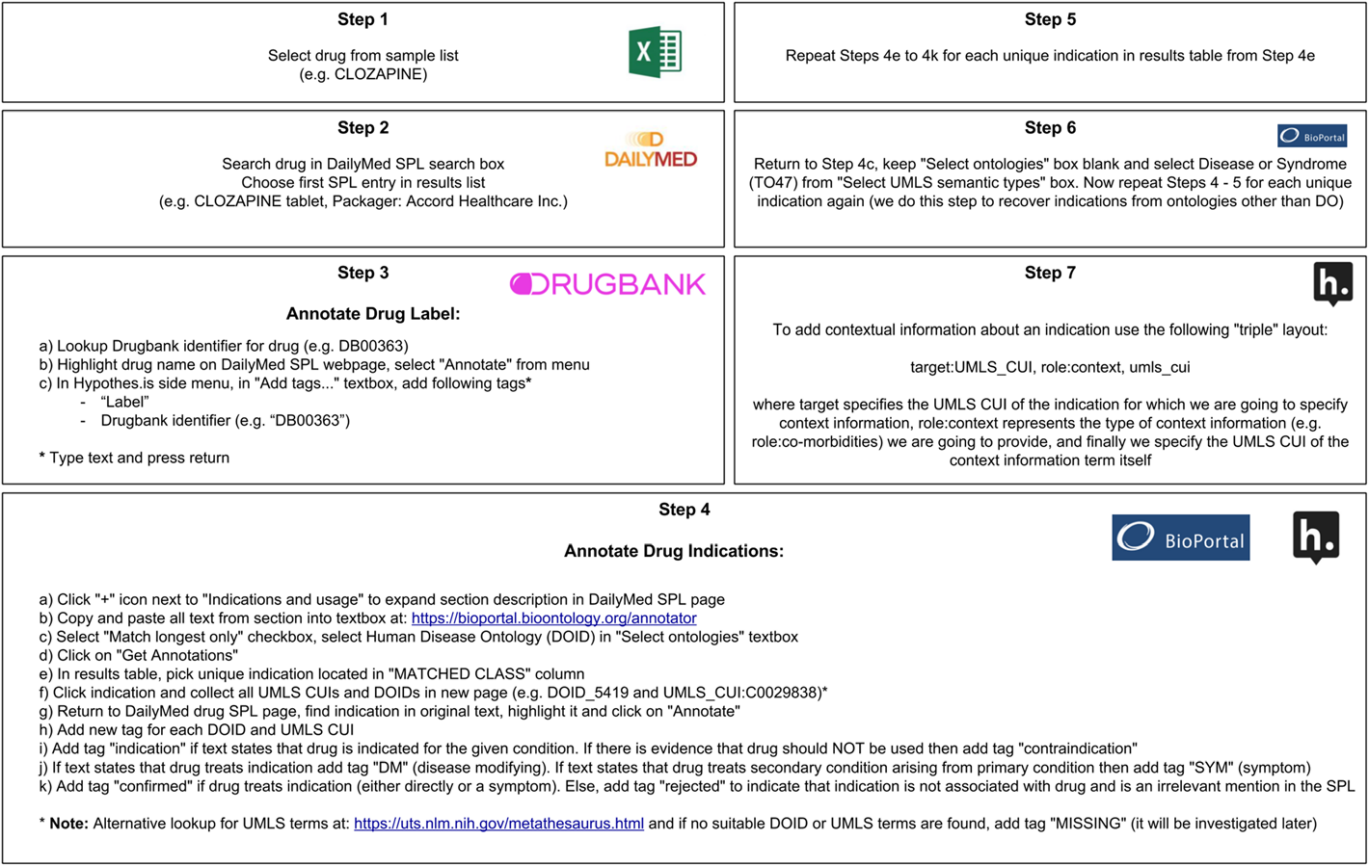
Drug indication curation protocol - outline of steps

We developed a protocol to systematize the process of recording the therapeutic usage annotations for the SPLs. A summary of the steps outlining this protocol is given in Fig. 3. We also provide full, detailed and reproducible protocol document (called “InContext Annotation Protocol.docx”) used by our annotators in our persistent repository: https://github.com/MaastrichtU-IDS/incontext-indications-analysis.

Essentially, for each drug, the task of the annotators can be divided into three main phases:

1. Identify all disease or possible indication or disease mentions in the DailyMed SPL for the drug,
2. Annotate the SPL with the Drugbank ID of the drug it is describing, as well as the Unified Medical Language System (UMLS) and DOID terms which describe all disease mentions appearing in the text (additionally distinguishing between indications and non-indications by annotating them with appropriate tags). The criteria for distinguishing between indications and non-indications in the text is to read the text in the SPL carefully to find any evidence to suggest that a particular disease mention is treated by the given drug or not. If there is evidence in phrasing of the text which clearly shows that the disease mention is *not* treated by the drug directly or *should not* be treated by the drug, then it is considered a non-indication. For example, in a DailyMed SPL for the antineoplastic drug Kymriah, a portion of the indications and usage section text states: “KYMRIAH is not indicated for treatment of patients with primary central nervous system lymphoma.” While automated named entity extraction algorithms might identify central nervous system lymphoma as a disease mention (and hence indication for the drug), it certainly does not represent an indication for the drug.
3. For each indication and non-indication in Phase 2., provide structured annotations for the contextual information appearing in the SPL, concerning the therapeutic use of the drug for that indication.

For Phase 1, we use the BioPortal Annotator to automatically extract disease mentions in the text. We configured the annotator to first recognize terms in the text from the Human Disease Ontology (DO) [33] – Step 4c. Thereafter, we configured it to recognize terms from other ontologies in BioPortal to capture diseases not mentioned in DO – Step 6. Critically, the curators also had to collect the codified identifiers for each indication in their respective ontologies, making them computable and interoperable. For this study we collected the identifiers for the indications from their mappings in the DO and the Unified Medical Language Services (UMLS) MetaThesaurus [34]. UMLS terms are codified using unique identifiers beginning with the letter “C” followed by seven single-digit numbers ranging from 0 to 9. These are called UMLS concept unique identifiers (CUIs) or UMLS CUIs. DO terms are codified with Disease Ontology identifiers (sometimes called DO-IDs). These begin with the letter sequence “DOID” followed by up to seven single-digit numbers uniquely identifying the disease under consideration.

**Fig. 4:**
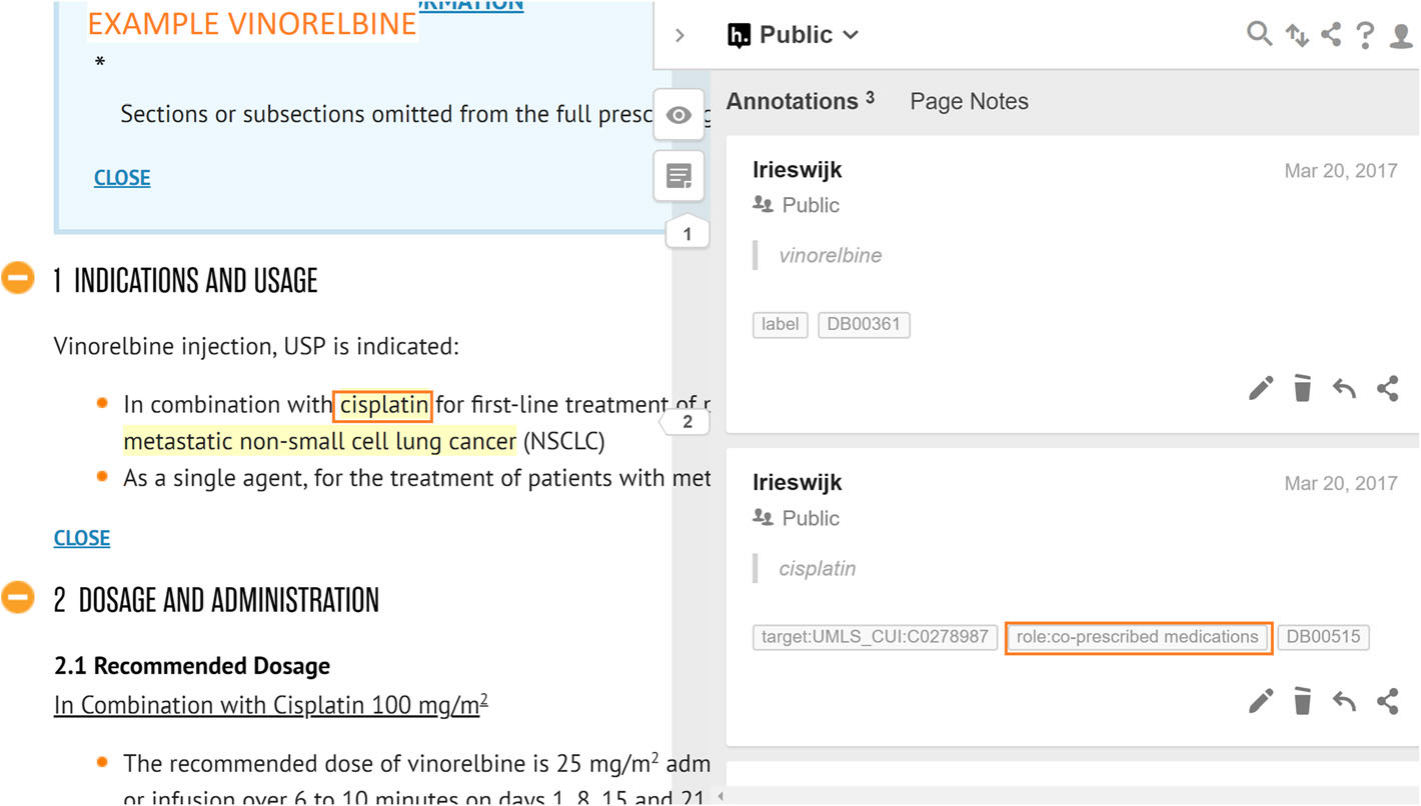
Example of medical context type - Vinorelbine - Co-prescribed medication

**Fig. 5:**
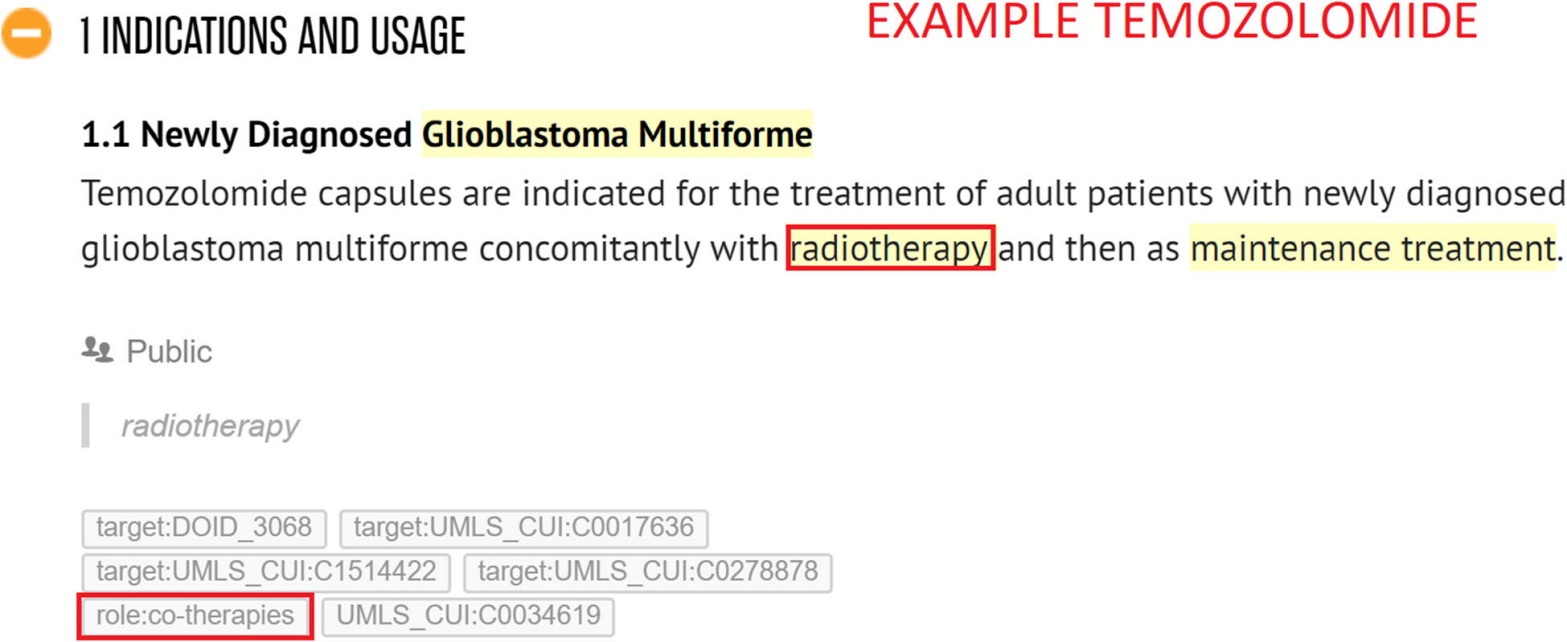
Example of medical context type - Temozolomide - Co-therapies

**Fig. 6:**
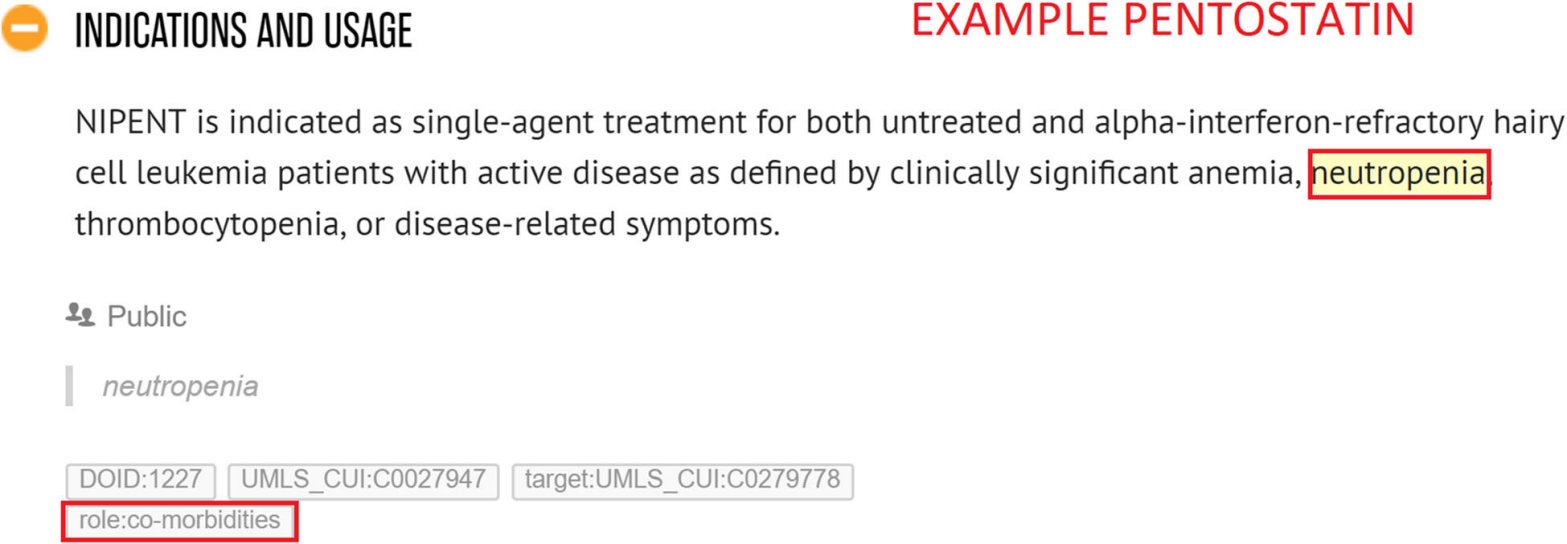
Example of medical context type - Pentostatin - Co-morbidities

**Fig. 7:**
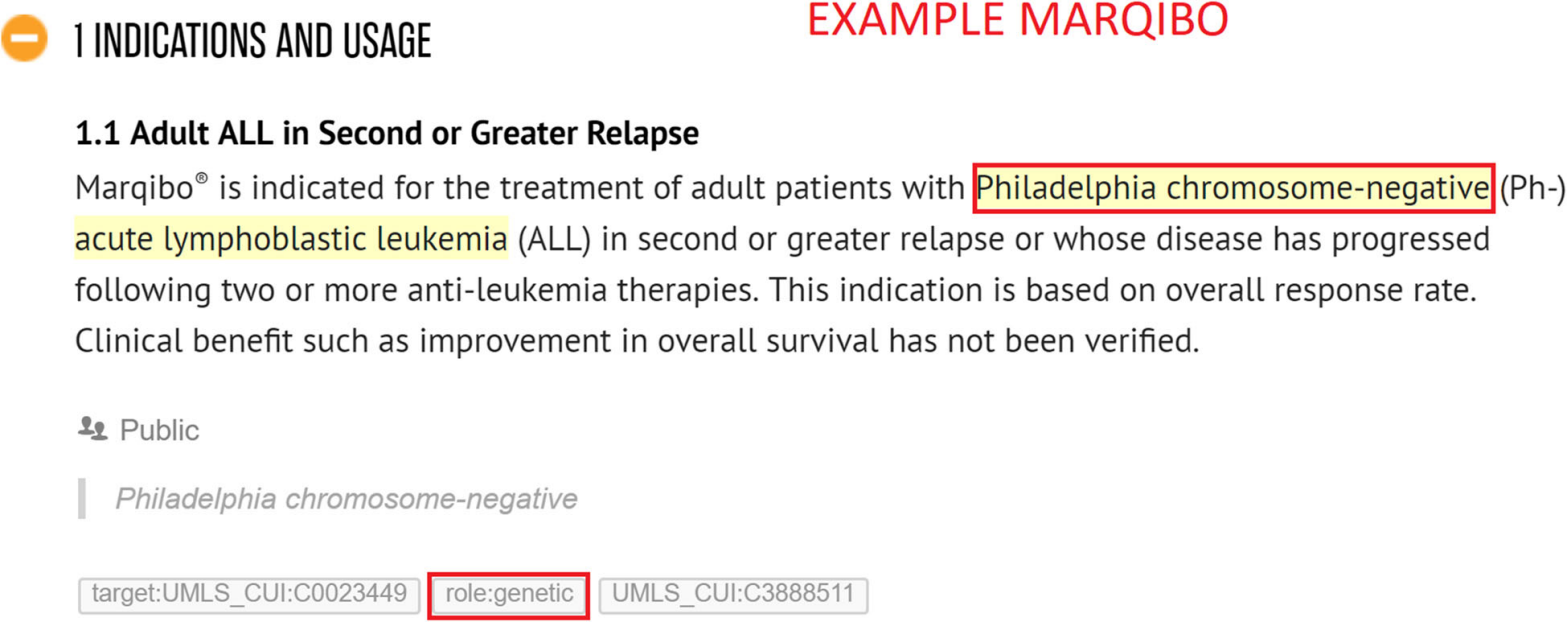
Example of medical context type - Marqibo - Genetics

**Fig. 8:**
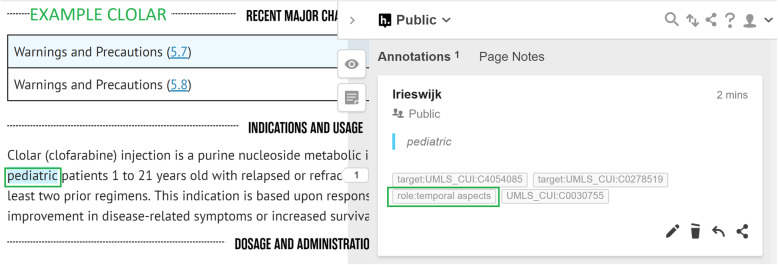
Example of medical context type - Clolar - Temporal aspects

For Phase 2, the annotators are given instructions how to discriminate between irrelevant disease mentions and actual drug indications in the protocol guidelines provided in the document “InContext Annotation Protocol.docx.docx” located in: https://github.com/MaastrichtU-IDS/incontext-indications-analysis. The same document provides clear instructions on how to use the Hypothes.is annotator to create the annotation tags specifying the indications on the relevant DailyMed SPL webpage. Finally, in Phase 3, the annotation guidelines document explains how to use the Hypothes.is tool to add other medical context information about the use of the drug. We distinguish between five categories of contextual information here:

1. *Co-prescribed medication:* drugs commonly prescribed together with the given drug. Often, in an SPLs for a particular drug, we find that indications are listed that should only be used in conjunction with another mentioned drug or class of drugs. For example, *Vinorelbine* is indicated to treat *metastatic non-small cell lung cancer* in combination with *Cisplatin* (see Fig. 4).
2. *Co-therapies:* procedures or therapies that should be applied in combination with the drug. Sometimes other (non-drug related) therapies should be administered to the patient along with the given drug. For example, *Temozolomide* should only be used in combination with *radiotherapy* to treat *newly diagnosed Glioblastoma Multiforme* (see Fig. 5)*.*
3. *Co-morbidities:* diseases or conditions that commonly occur together (with a target condition) in the same patients. It is important to know what other diseases or conditions commonly co-occur so that a physician can ascertain whether the different medications that may be used to treat them interact with each other favourably (see Fig. 6).
4. *Genetics:* particular genetic strains of a disease. For example, *Marqibo* is indicated to treat patients with *Philadelphia chromosome-negative (Ph-) acute lymphoblastic leukemia* (see Fig. 7).
5. *Temporal aspects:* information which explains at what life stage, disease stage, or treatment phase a drug should be administered. This can be a life stage such as pregnancy or an age group (e.g. 12–24 years) etc. For example, *Clolar* should only be administered to pediatric patients from 1 to 21 years old (see Fig. 8).

**Fig. 9:**
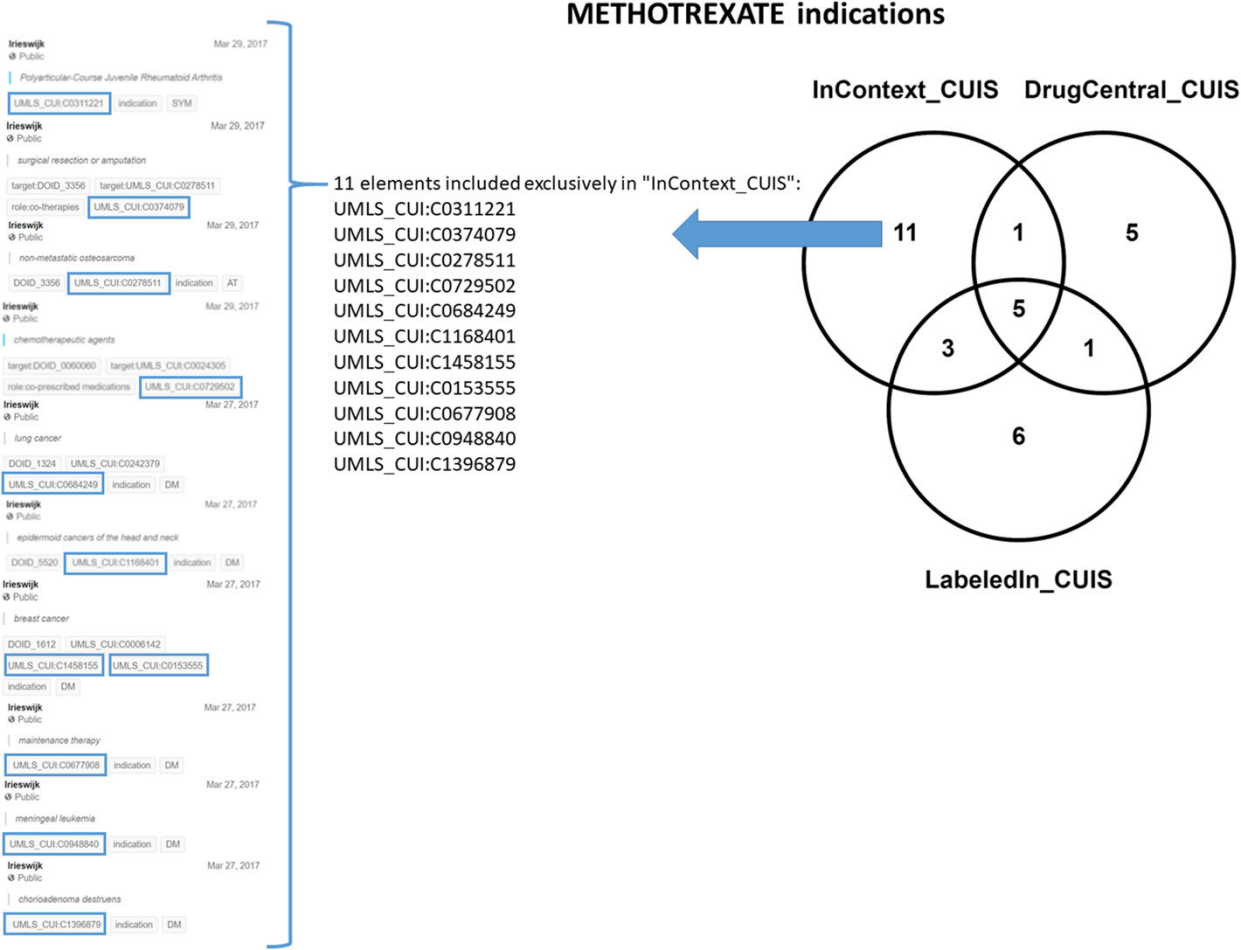
Example of a drug - Methotrexate - for which InContext provides 11 unique indications that do not appear either in DrugCentral or LabeledIn

Whenever an annotator captures contextual information, they highlight either the name of the co-therapy, co-prescribed medication, co-morbidity, genetic strain, or piece of temporal information in Hypothes.is. Thereafter, they assign a “role” tag to the highlighted information which indicates the type of contextual information. This will be one of “role:co-therapies”, “role:co-prescribed medication”, “role:co-morbidities”, “role:genetic” or “role:temporal aspects”. Additionally, the MetaThesaurus/ontology term of the highlighted entity will be looked up by the annotator in the UMLS and / or Disease Ontology and added as another tag to the highlighted information (if there exists such a term). As mentioned in the examples in Figs. 4, 5, 7, 8, and 9, it can be appreciated that the kinds of contextual information we capture are potentially vital for a physician to take into account when administering the drug of interest to the patient. The information is also essential for drug repurposing algorithms to consider when making predictions about other uses for drugs. The categories of contextual information we proposed in this section are representative of the main types that we encountered when curating indications for anti-cancer and cardiovascular drugs from DailyMed SPLs. While this list may not be exhaustive, it represents a useful ontological basis from which to develop a formal model to capture therapeutic intent of drugs.

### InContext corpus

The InContext corpus generated from the curation procedure outlined in Sections 3.1–3.3 is publicly available directly via the Hypothes.is browser plugin. This means that any user who has the browser plugin installed can view and extract the annotations directly in their browser by browsing the SPL webpages for drugs on the DailyMed website. To help with integrating our corpus into existing software and computational analysis workflows, Hypothes.is also has a public application programming interface (API) to enable software developers to programmatically access and extract the InContext annotations [27].

As mentioned in Sections 3.1–3.3, the corpus annotates two types of information - indication labels and medical context for these indications. Both types of information can be accessed via the Hypothes.is browser plugin and through the API. As an alternative mode of access for the indication labels only is provided in comma separated value (CSV) format from the following Github repository: https://github.com/MaastrichtU-IDS/incontext-indications-analysis. This files in this repository also provide a more detailed statistical overview of the number of indications curated overall and per drug.

### Expert reclassification of indications

To disambiguate disease terms, we asked two medical experts to give their opinion on the equivalence of all pairs of indications with distinct UMLS CUIs (per drug across the drug information resources). Our experts have diverse backgrounds as medical professionals, and are located in different institutions on separate continents: one is embedded in the Maastricht University Medical Center (MUMC) in the Netherlands, and the other in the University of New Mexico’s Department of Internal Medicine, in the USA. We would ideally like at least three medical experts to identify indications pairs with different UMLS terms that are actually synonymous (or similar). We attempted to recruit five medical experts across three institutions but, due to demanding schedules, only two were available and willing to participate in the study. Disagreements between two experts would be difficult to resolve without a third party. Therefore, we chose the following setup for the task: we split the indication pairs into two equally sized sets and we assigned each set to be analyzed independently by one of the experts. Therefore, there was no need to handle disagreements. While this is not an ideal situation, we believe the results are still trustworthy. While the use of one expert would have raised doubts about bias, incorporating the opinion of two experts from different settings, in this way, mitigates bias and prevents one expert’s opinion from outweighing the other in the overall analysis. The experts were presented all relevant indication pairs in a spreadsheet and were asked to record “y”, “n” or “s” beside each pair if they determined that it represented “equivalent”, “dissimilar” or “similar” indications respectively. “Synonymous” indication terms are those that are distinct but refer to precisely the same disease or condition. Excluding synonymous pairs, the remaining pairs may possibly be regarded as “similar” to varying degrees. The experts were asked to comply with the following definition: two indications should be judged as “similar” if they appear not more than 2 edges away from each other in a taxonomy that classifies diseases and conditions in the cancer or cardiovascular domain (formulated from the medical expert’s own expertise and experience). However, when a condition leads to, or stems from, another condition we do not necessarily consider these as similar, nor do we regard co-morbidities as similar. We then used the analysis by the medical experts to refine our overlap numbers.

## Results

In order to gain preliminary insight into the overlap of InContext indications with other prominent indication databases, we decided to compare it with two leading examples - DrugCentral and LabeledIn. In this section, we present the methodology and results of the analysis. The central idea behind the comparison is that in InContext, we only count indications that are tagged or confirmed to be indications by our annotators who have read the contextual information. That is, we filter out irrelevant disease mentions as non-indications (see Section 3.3). The key point to realise here is that databases curating indications, even partially, through automated algorithms (e.g. DrugCentral and LabeledIn) will not have full control over the precision and recall of the indications. The LabeledIn protocol, for example, uses an algorithm to first extract all the disease mentions in the text. Thereafter, human annotators sort these mentions either as indications or non-indications. The problem here is that it cannot be known a priori if the algorithm will not miss any disease mentions in the text. The only reliable way to spot all disease mentions is to have human annotators carefully read the drug label manually. The InContext protocol employs this strategy at the expense of time-efficiency in curation. Therefore, we are actually measuring how reliably the semi-automated methods for curating indications in DrugCentral and LabeledIn capture FDA-approved indications for drugs (as curated manually in InContext). The question we try to answer here is, quantitatively speaking, “what is the level of agreement of the three databases overall?”

### DrugCentral

DrugCentral’s indications for the same set of sample drugs can be browsed by general users on their website, and for convenience, we also listed relevant information about these drugs in our Github repository. We separated the overlap analysis of DrugCentral and InContext into two parts: 1) a naïve count of overlapping indications for each candidate drug and a summative total of these (by counting unique ontology terms across the two sets for each drug), and 2) a revised count by first asking medical experts to verify if indications with different ontology terms actually constitute distinct indications (or whether they are effectively synonymous).

#### Comparison

**Fig. 10:**
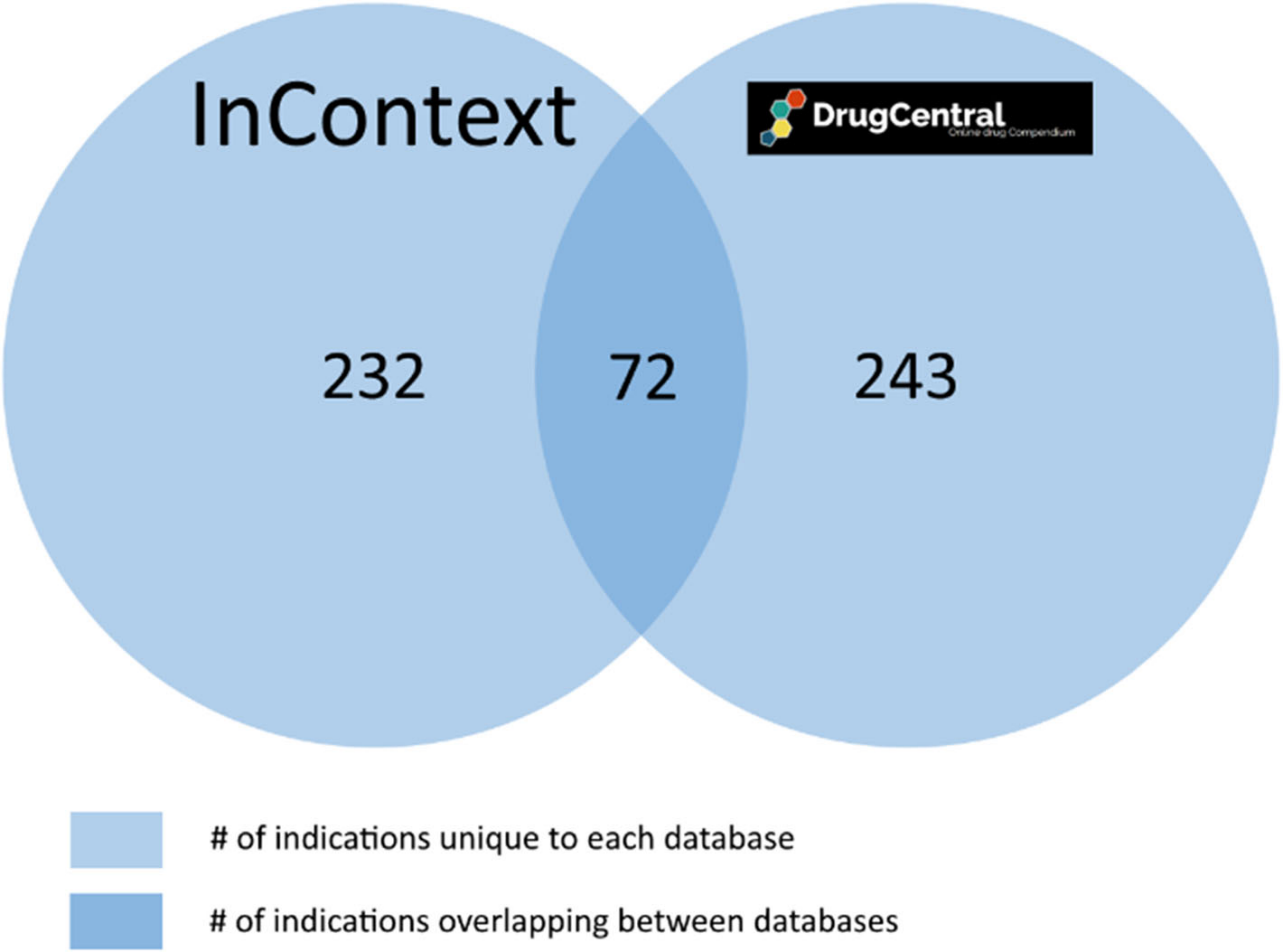
Initial overall overlap of indications between DrugCentral and InContext. *Legend:* The total number of overlapping ontology terms (representing indications) between DrugCentral and InContext, before any analysis of the veracity of these term mappings

**Fig. 11:**
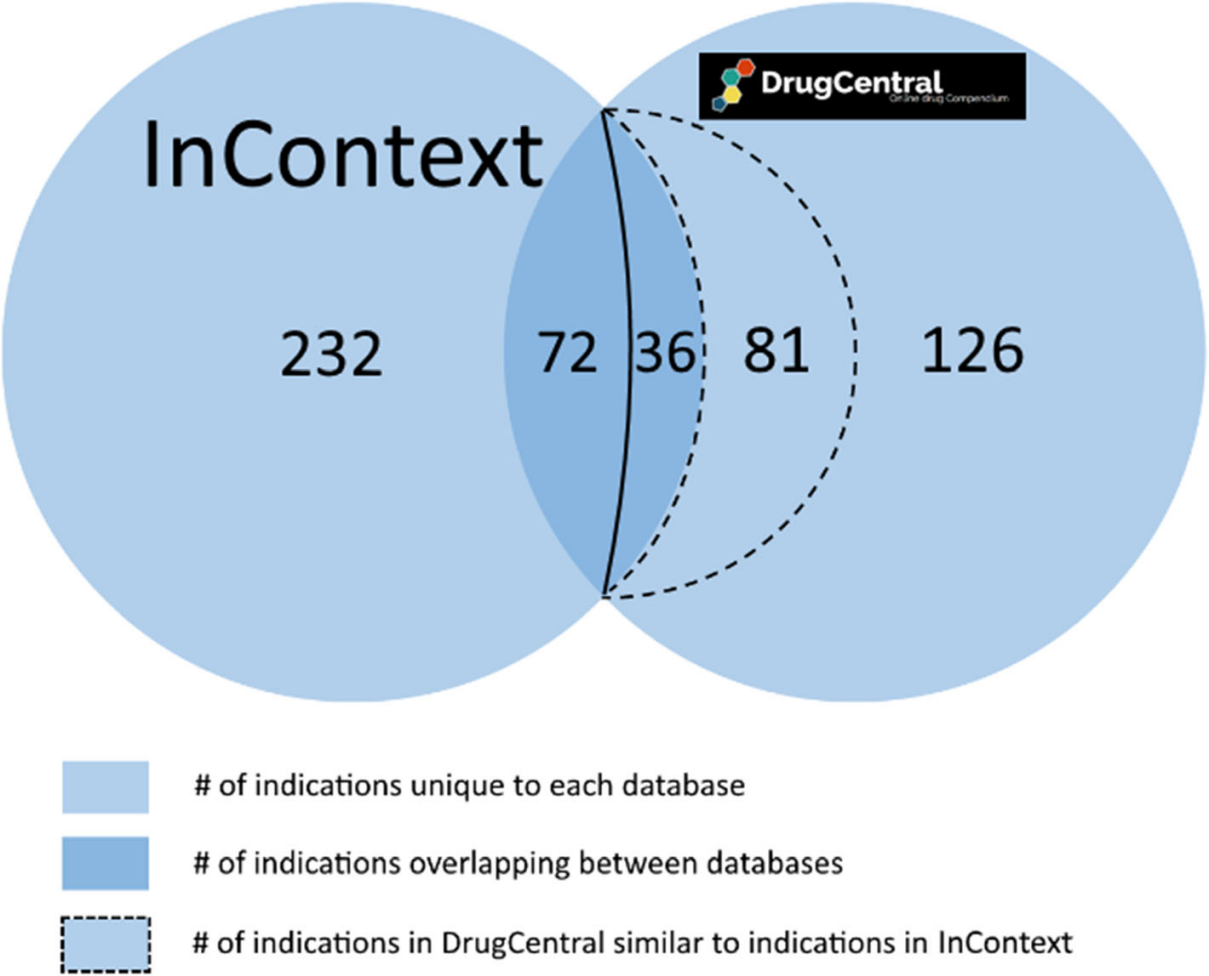
Overall overlap of indications after medical expert reclassification. Legend: Total number of overlapping indications between DrugCentral and InContext after medical experts identified indication pairs that are equivalent, but have different ontology terms. The number in the dotted region of the figure indicates the number of indications reclassified into the overlap

For each drug in our sample set of 158 anti-cancer and cardiovascular drugs, we compare each DrugCentral indication for this drug to those in InContext and determine the intersection or overlap of indications. We calculate the overlap by counting the number of UMLS CUIs or DOIDs (representing indications) that overlap between the two resources for that drug. Figure 10 illustrates the number of UMLS CUIs which intersect between DrugCentral and our curated indication set. Approximately 58% of our sample drugs belong to the cardiovascular class and the remaining 42% belong to the anti-cancer class. 54% of the overlapping indications are for the cardiovascular drugs and the other 46% are for the antineoplastic drugs. On average, 15.6% of the indications for each antineoplastic drug in DrugCentral had a match to one from our reference set. This number rises to 23% for the cardiovascular drugs. Although this number is surprisingly low, many non-overlapping indications were later reclassified into the overlap following re-evaluation by medical experts. A naïve comparison is obviously too coarse to give an insightful picture into the overlap. This is because, at least in some cases, a DrugCentral indication may not share the same UMLS CUI as the same (or highly similar) indication from InContext. For example, *Hyperaldosteronism* and *Conn’s syndrome* are synonymous although having distinct UMLS CUIs (a similar issue arises for *Wegener’s granulomatosis* and *Granulomatosis with polyangiitis*). A converse problem arises with *Insomnia* and *Sleeplessness.* In this case they are different conditions, but they are mapped to the same UMLS CUI. DrugCentral shows correctly that *Diphenhydramine* is indicated for sleeplessness. However, because UMLS maps sleeplessness to insomnia, it can be misleadingly inferred that these two conditions are the same. This is not a shortcoming of DrugCentral, but of the imprecise definition of terms in the vocabulary. Therefore, in order to address this issue, we employed the help of medical experts to classify and disambiguate synonymous and distinct indications across InContext and DrugCentral for the sample drugs (see Section 3.5). Figure 11 represents the overlap numbers after reclassification by the medical experts.

The number of DrugCentral indications previously not matched to InContext indications almost halves after expert reclassification. Approximately 34% of the indications for each drug can now be mapped to indications in InContext, while 81 non-overlapping DrugCentral indications are similar, according to the experts, to indications in InContext.

#### Discussion

Approximately 45% of the DrugCentral indications for each drug can be mapped to indications in InContext. In all our analyses there remains at least a third of DrugCentral’s indications which remain unsupported by our reference set of indications. However, there are some considerations that should be taken into account relating to data quality.

Firstly, “Indications in DrugCentral were collated from OMOP vocabularies [35] for those drugs approved before 2012; for drugs approved after 2012, indications were extracted from drug labels, and mapped to SNOMED-CT [36] concepts. Indication data from these two sources are currently being harmonized using the UMLS application programming interface, as well as manual mapping” [7]. This means that indications approved prior to 2012 may not have been mapped to ontology terms like SNOMED-CT. Whereas this indicates awareness of the shortcoming, it further implies that any commercial or public system relying on OMOP vocabularies are likely to have the same shortcoming unless indications are processed post-integration. In our DrugCentral dataset we found that roughly 22% of the indications were from this OMOP set (they did not have UMLS CUIs or any ontology term identifiers that could be mapped to UMLS terms). For example, five out of six indications for *Tretinoin* were missing mappings to ontology terms, and could not be mapped to UMLS CUIs using the UMLS MetaThesaurus [37]. Since we only analysed indications that had UMLS CUIs, all other indications were not considered in this study.

Secondly, in many cases, indications for drugs can vary drastically according to the specific formulation (dosage forms and strength) of the drug product. In DailyMed, indications for the same core drug can be spread across SPLs for different *products* containing this drug. For example, *Tretinoin* is indicated for *Acne Vulgaris* in some formulations and for *Acute Promyelocytic Leukemia* in others. Another example is that, when combined with *Acetaminophen* 500 mg, *Diphenhydramine* 25 mg is indicated as a “sleep aid”, as formulated in *Tylenol PM*. This was noted as a side-effect for the 25 mg formulation of *Diphenhydramine* in *Benadryl*, an anti-histamine.

In the InContext protocol we selected only the *first* product in the list of results (Step 2 of Fig. 3), while in DrugCentral it appears that indications are collected across multiple formulations of a drug. Therefore, DrugCentral will, in general, source more indications than InContext. However, this does not mean that more indications are “better”. The indications should be accurate (and FDA-approved). Whether it is more suitable to source indications from multiple SPLs for a drug, or just a single canonical SPL, depends on the drug repositioning application one is interested in.

However, there are exceptions to the rule that DrugCentral finds more indications. In fact, there are some cases where our curated indications are supersets of the indications in DrugCentral, for a particular drug. For example, *Reserpine* is purported to treat *Psychotic disorder* and *Severe Hypertension* in DrugCentral, which is corroborated by our curated database. Our curated database also mentions *Schizophrenia* as an additional indication for *Reserpine* which does not appear in DrugCentral.

### LabeledIn

LabeledIn [24] is a large scale effort to curate drug-disease associations from text. Similar to InContext, LabeledIn uses DailyMed SPLs as its curation source and its original version is a purely manual effort by human annotators. However, apart from being a much larger database of indications for over 8000 drugs, LabeledIn differs from InContext in two key respects. Firstly, it does not curate any precise contextual information about its listed indications. Secondly, in some cases, LabeledIn curates indications from multiple product formulations (and hence SPLs) containing the same drug. InContext, on the other hand, sources indications from one SPL (Fig. 3, Step 2).

This latter point makes a direct comparison of indication overlap between the resources untenable. However, we can still compare indication overlap for cases where both resources curate from the same SPL. There are only 11 drugs that both LabeledIn and InContext provide indications for. These SPLs are for: *Nimodipine, Bortezomib, Methotrexate, Bevacizumab, Pemetrexed, Cetuximab, Dronedarone, Sorafenib, Rituximab, Trastuzumab* and *Propafenone.* The number of indications for each drug SPL, and the overlap numbers, are indicated in Table 2.

**Table 2:** Indication overlap numbers for LabeledIn and InContext

Drug Name	#ic	#dc	#li	#all	#ic_dc	#ic_li	#dc_li
Nimodipine	1	1	1	0	0	0	0
Bortezomib	1	2	1	0	1	0	1
Methotrexate	20	12	16	5	6	8	6
Bevacizumab	9	8	1	0	2	0	0
Pemetrexed	4	2	1	0	1	0	0
Cetuximab	6	5	1	0	0	0	0
Dronedarone	1	2	3	0	0	1	1
Sorafenib	5	3	1	1	1	1	1
Rituximab	6	5	8	2	2	2	5
Trastuzumab	5	3	1	0	1	1	0
Propafenone	4	4	5	1	2	2	1

**#ic, #dc, #li**: Number of indications for the given drug SPL in InContext, DrugCentral and LabeledIn respectively

**#all, #ic_dc, #ic_li, #dc_li**: Number of indications for the given drug SPL that overlap in all three databases, between InContext and DrugCentral, between InContext and LabeledIn, and between DrugCentral and LabeledIn, respectively

**Fig. 12:**
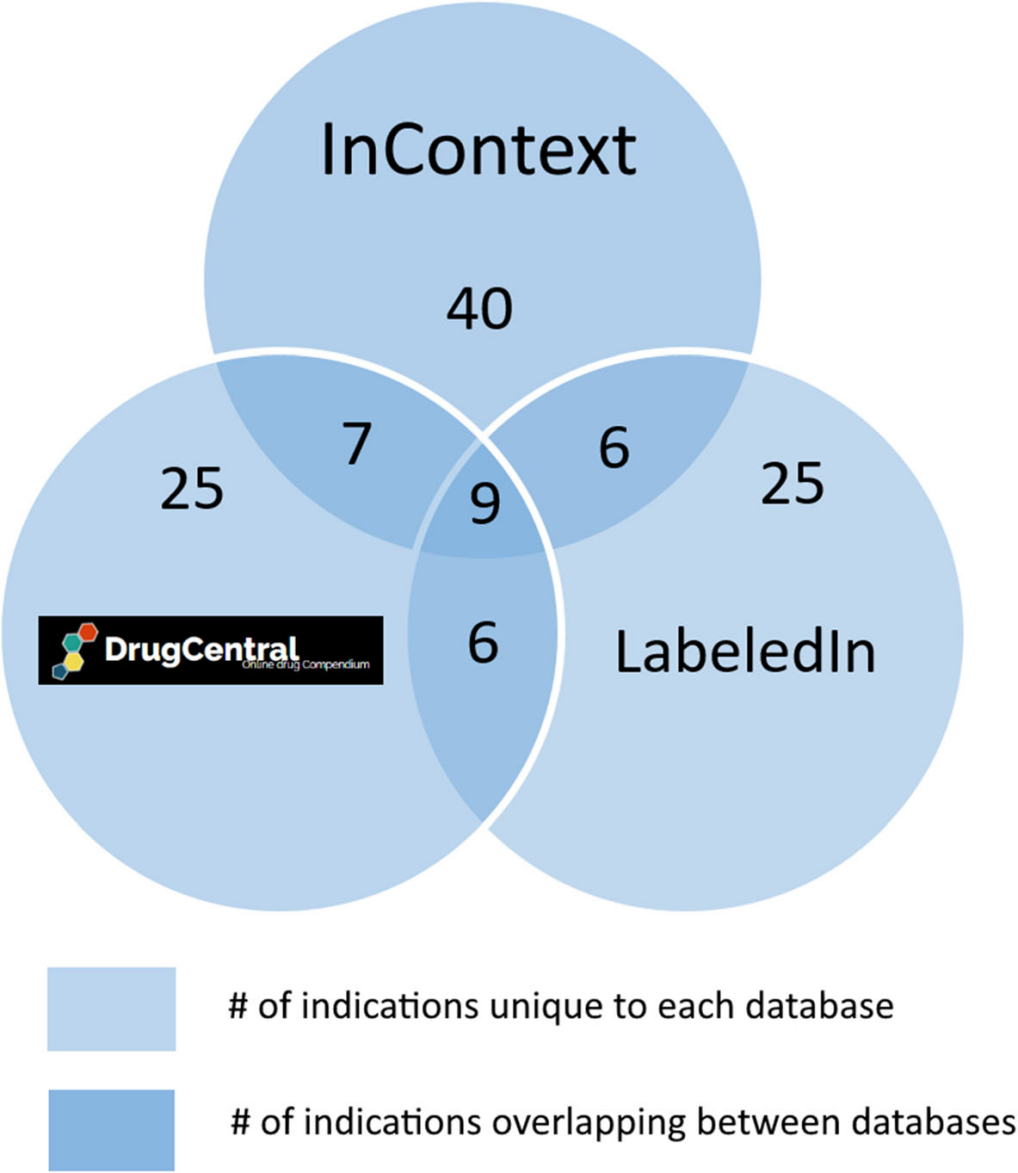
Overlap of indications between DrugCentral, InContext and LabeledIn for common SPLs. Legend: The number of overlapping indications between DrugCentral, LabeledIn and InContext for drug labels that all three resources curate or document

To analyse actual indication terms for the SPLs in Table 2 and to retrieve the unique terms for the different databases, the reader can consult our dataset: https://github.com/MaastrichtU-IDS/incontext-indications-analysis, and in particular, the “LabeledIn_comparison_results_table.csv” file. An interesting observation from Table 2 is that, for 7 out of 11 SPLs, no indications can be corroborated by all three resources. In other words, the finding is that there is absolutely no overlap in indications between LabeledIn, DrugCentral & InContext, for 7 out of the 11 SPLs that are covered by both LabeledIn and InContext. This is interesting because one might hope that resources purporting to provide accurate drug indication association information should agree, at least to some extent, on these associations. Figure 12 provides a summary of the number of indications overlapping for drugs that all three resources cover. An example demonstrating some data quality issues highlighted in this kind of study, is the SPL for *Nimodipine*. The drug is indicated for *Subarachnoid intracranial hemorrhage* by both InContext and DrugCentral but the DrugCentral database provides an imprecise UMLS CUI for this indication - C0038525 (which refers to the more general *Subarachnoid hemorrhage*). The LabeledIn entry for the same SPL asserts that *Berry Aneurysm* is the correct indication for *Nimodipine* and, examining the SPL on DailyMed, we find the following clue to its usage: “… reducing the incidence and severity of ischemic deficits in patients with subarachnoid hemorrhage from ruptured intracranial berry aneurysms …” This can be interpreted in multiple ways but what is clear is that each database captures part of the information for the full precise indication. This example clearly illustrates a problem with unstructured representations of indication information. Not knowing precise indications for drugs can clearly affect the accuracy of computational algorithms in drug repurposing and motivates for more structured representations of such information, as stated elsewhere [11].

There are a few cases in Table 2 where LabeledIn provides more indications for a drug (3 out of 11 cases) than InContext, but in the majority of cases (8 out of 11 times) the converse is true. Figure 6 gives an overview of the degree of overlap in the three resources for the common SPLs documented in Table 2. Figure 9 represents an example of *Methotrexate* where InContext provides 11 unique indications that do not appear in DrugCentral and LabeledIn. This unique information contains indications where the drug is applied to a specific medical context. (i.e. indications tagged with “role:co-therapies”, “role:co-prescribed medications” or “role:co-morbidities”). Additionally, this information captured by InContext also represents other disease modifying (DM) as well as symptomatic (SYM) indications. Since the overlap of commonly annotated SPLs between InContext and LabeledIn is so low, a more in depth analysis would likely not reveal more significant insights.

## Conclusions and future directions

We have established a reference set of FDA-approved indications for approximately 150 antineoplastic and cardiovascular drugs which also include contextual information about the therapeutic intent of drugs, called InContext. InContext extends the state of the art in drug indication curation from text by adding therapeutic context information about the usage of drugs. There have been no such efforts in the literature, to date. InContext is thus an important and novel contribution to the body of work on the informatics of drug indication and therapeutic use. Contextual information is crucial in prescribing the right treatments and therapeutic guidelines for patients. One of the main findings in this work is that the overlap in drug indications found in FDA-approved SPLs and those found in existing reputable drug resources that partially curate their indications using automated methods (e.g. DrugCentral) is significantly lower than what is required for precision in silico drug repurposing. LabeledIn is a human curation effort which avoids some pitfalls of automated extraction, but it does not record in detail the reasons for identifying or rejecting disease mentions as indications. We argue that understanding and formalising these reasons, and classifying them into instructive categories of information about therapeutic intent, is an important step towards improving the accuracy of drug indication extraction and the level of agreement between drug indication corpora. InContext represents a novel step in the direction towards formalising and capturing these reasons for classifying drug indications accurately in drug labels.

Having a dataset like InContext, that is enriched beyond drug-indication pairs, is also invaluable for augmenting clinical decision support systems, and for strengthening data-driven applications in precision medicine such as drug repurposing. It is essential for the computational drug repurposing community to be aware of such datasets, and to support their development so that repurposing algorithms could benefit from them. The threshold for acceptable data quality for drug repurposing is high. Machine Learning has been a successful tool for new discoveries in drug repositioning, but its predictions (outputs) are only as good as the quality of its inputs (the data). Setting up and conducting clinical trials to test new indications for existing drugs is costly and one would ideally hope that computationally predicted drug-indication pairs are based on sound data, to increase the chances of success of these trials.

For our sample of 150 anti-cancer and cardiovascular drugs, we have calculated an overall level of agreement that InContext has with a leading online drug compendium (DrugCentral) and a state-of-the-art medication-indications database (LabeledIn). We found that roughly 40% of indications on DrugCentral could not be corroborated by InContext (23%, respectively, for LabeledIn). A more in depth analysis is needed to draw any claims about the quality of the extracted drug indications pairs. What remains clear is that, in all studied databases, there is significant scope for improvement of accuracy and precision of therapeutic intent information for drugs.

This highlights one of the fundamental issues in medicine: that lacking a quantitative language “is the flaw of biological research” [38]. Indeed, developing a system to precisely map drug indications remains one of the desiderata for computational drug repurposing. Our future endeavors will be to extend our analysis to more drugs in the classes investigated here, to explore the use of crowdsourcing to scale our curation tasks, to extend our annotation protocol to collect additional types of medical context information and, finally, to assess and improve the data quality of information extracted using the InContext protocol.

## Data Availability

The datasets supporting the conclusions of this article are available in the following Github repository, [https://github.com/MaastrichtU-IDS/incontext-indications-analysis].

## Abbreviations

CUI: Concept Unique Identifier
SNOMED CT: Systematized Nomenclature Of MEDicine Clinical Terms
UMLS: Unified Medical Language System
NLP: Natural Language Processing
FDA: United States Food and Drug Administration
SPL: Structured Product Label
NCBO: National Center for Biomedical Ontologies
API: Application Programming Interface
DO: Disease Ontology
DOID: Disease Ontology Identifier
OMOP: Observational Medical Outcomes Partnership
NER: Named Entity Recognition

## References

[1] DiMasi JA, Grabowski HG, Hansen RW (2016). Innovation in the pharmaceutical industry: new estimates of R&D costs. J Health Econ.

[2] Lamberti MJ, Getz KA. Profiles of new approaches to improving the efficiency and performance of pharmaceutical drug development. In: Tufts CSDD White papers: Tufts Center for the Study of Drug Development; 2015. https://csdd.tufts.edu/s/CSSD_PhRMAWhitePaperNEWEST2.pdf. Accessed 10 Dec 2017.

[3] Gottlieb A, Stein GY, Ruppin E, Sharan R (2011). PREDICT: a method for inferring novel drug indications with application to personalized medicine. Mol Syst Biol.

[4] Wei WQ, Cronin RM, Xu H, Lasko TA, Bastarache L, Denny JC (2013). Development and evaluation of an ensemble resource linking medications to their indications. J Am Med Inform Assoc.

[5] Wishart DS, Knox C, Guo AC, Shrivastava S, Hassanali M, Stothard P, Chang Z, Woolsey J (2006). DrugBank: a comprehensive resource for in Silico drug discovery and exploration. Nucleic Acids Res.

[6] Avram S, Bologa CG, Holmes J, Bocci G, Wilson TB, Nguyen DT, Curpan R, Halip L, Bora A, Yang JJ, Knockel J, Sirimulla S, Ursu O, Oprea TI. DrugCentral 2021 supports drug discovery and repositioning. Nucleic Acids Research. 2021;49(D1):D1160–9. 10.1093/nar/gkaa997. Accessed 08 Jan 2021. 10.1093/nar/gkaa997 PMC7779058 33151287

[7] Ursu O, Holmes J, Knockel J, Bologa GC, Yang JJ, Mathias SL, Nelson SJ, Oprea TI (2017). DrugCentral: online drug compendium. Nucleic Acids Res.

[8] Névéol A, Lu Z. Automatic integration of drug indications from multiple health resources: ACM IntHealth Informatics Symposium; 2010. 10.1145/1882992.1883096.

[9] Fung KW, Jao CS, Demner-Fushman D (2013). Extracting drug indication information from structured product labels using natural language processing. J Am Med Inform Assoc.

[10] Dogan RI, Lu Z (2012). An improved corpus of disease mentions in PubMed citations. Proceedings of the Workshop on Biomedical Natural Language Processing.

[11] Nelson SJ, Oprea TI, Ursu O, Bologa CG, Zaveri A, Holmes J, Yang JJ, Mathias SL, Mani S, Tuttle MS, Dumontier M (2017). Formalizing drug indications on the road to therapeutic intent. J Am Med Inform Assoc.

[12] Leaman R, Rezarta Islamaj D, Lu Z (2013). DNorm: disease name normalization with pairwise learning to rank. Bioinformatics.

[13] Khare R, Jiao L, Lu Z. Toward creating a gold standard of drug indications from FDA drug labels: Healthcare Informatics (ICHI); 2013. 10.1109/ICHI.2013.11.

[14] DailyMed entry for Rituximab. 2019. https://bit.ly/2l1D6sk. Accessed 21 July 2019.

[15] Calixto NM, Dos Santos DB, Bezerra JCB, Silva LA (2018). In silico repositioning of approved drugs against Schistosoma mansoni energy metabolism targets. PLoS One.

[16] Roberts DM, Gallapatthy G, Dunuwille A, Chan BS (2016). Pharmacological treatment of cardiac glycoside poisoning. Br J Clin Pharmacol.

[17] DailyMed entry for Alprostadil. 2018. https://goo.gl/o16qmf. Accessed 10 June 2018.

[18] DailyMed. 2018. http://dailymed.nlm.nih.gov. Accessed 10 June 2018.

[19] Khare R, Burger JD, Aberdeen JS, Tresner-Kirsch DW, Corrales TJ, Hirchman L, Lu Z. Scaling drug indication curation through crowdsourcing. Database. 2015. 10.1093/database/bav016. 10.1093/database/bav016 PMC4369375 25797061

[20] Leaman R, Lu Z (2016). TaggerOne: joint named entity recognition and normalization with semi-Markov models. Bioinformatics.

[21] Zheng L, Yumak H, Chen L, Ochs C, Geller J, Kapusnik-Uner J, Perl Y (2017). Quality assurance of chemical ingredient classification for the National Drug File–Reference Terminology. J Biomed Inform.

[22] Kuhn M, Letunic I, Jensen LJ, Bork P (2015). The SIDER database of drugs and side effects. Nucleic Acids Res.

[23] Salmasian H, Tran TH, Chase HS, Friedman C (2015). Medication-indication knowledge bases: a systematic review and critical appraisal. J Am Med Inform Assoc.

[24] Khare R, Jiao L, Lu Z (2014). LabeledIn: cataloging labeled indications for human drugs. J Biomed Inform.

[25] Khare R, Wei CH, Lu Z (2014). Automatic extraction of drug Indications from FDA drug labels. AMIA Annu Symp Proc.

[26] Hypothes.is. https://web.hypothes.is. Accessed 10 June 2018.

[27] Hypothes.is API. https://h.readthedocs.io/en/latest/api. Accessed 10 June 2018.

[28] BioPortal Annotator. https://bioportal.bioontology.org/annotator. Accessed 10 June 2018.

[29] BioPortal. http://bioportal.bioontology.org. Accessed 10 June 2018.

[30] Aronson AR, Lang FM (2010). An overview of MetaMap: historical perspective and recent advances. J Am Med Inform Assoc.

[31] Stewart SA, Von Maltzahn ME, Abidi SSR (2012). Comparing Metamap to MGrep as a tool for mapping free text to formal medical lexicons. Proceedings of the international workshop on knowledge extraction & consolidation from social-media in conjunction with the international semantic web conference.

[32] BioPortal DO. https://bioportal.bioontology.org/ontologies/DOID. Accessed: May, 2017.

[33] Schriml LM, Arze C, Nadendla S, Chang YW, Mazaitis M, Felix V, Feng G, Kibbe WA (2012). Disease ontology: a backbone for disease semantic integration. Nucleic Acids Res.

[34] Nelson SJ, Powell T, Srinivasan S, Humphreys BL (2009). The unified medical language system (UMLS) project. Encycloped Library Inform Sci.

[35] OMOP vocabularies. https://www.ohdsi.org/data-standardization/vocabulary-resources. .

[36] Donnelly K (2006). SNOMED-CT: the advanced terminology and coding system for eHealth. Stud Health Technol Inform.

[37] UMLS MetaThesaurus. https://uts.nlm.nih.gov/metathesaurus.html. Accessed: May, 2017.

[38] Lazebnik Y (2002). Can a biologist fix a radio? — or, what I learned while studying apoptosis. Cancer Cell.

